# Correlation Between *in Vivo *Accumulation and *in Vitro *Adhesion of Liver associated Lymphocytes in and Around Liver Adenocarcinoma Metastases

**DOI:** 10.1186/1476-5926-2-S1-S55

**Published:** 2004-01-14

**Authors:** Marek Stanczyk, Waldemar Olszewski, Sergiusz Durowicz, Marek Maruszyński

**Affiliations:** 1 Central Clinical Hospital of Military Medical School, Warsaw, Poland; 2 Department of Surgical Research & Transplantology, Medical Research Center, Polish Academy of Science, Warsaw, Poland

## Introduction

Liver is a major site for formation of metastases. The local immune function of liver is associated with a specific mononuclear cells population transiently retained in the liver, originating most probably from blood. Liver mononuclear cells consist mainly of natural killer (NK) cells, T gamma delta cells and T cells expressing NK molecules. The latter are found in low proportions in peripheral blood [1-3]. Tumoricidal activity requires cell to cell contact initiated by functionally relevant adhesion molecules. It is not clear which adhesion molecules are responsible for the site directed traffic and binding of mononuclear cells within the tumor microenvironment and which populations of mononuclear cells reveal prediction to accumulate around and within the tumor tissue during postextravasation phase of tumor growth [4-6]. Defining these populations and molecules may help to elucidate the mechanisms that guide mononuclear cells to and within the tumor microenvironment. This may be important for two reasons: first evaluation of their cytotoxic capacity, second isolation and culturing cells for transfection with cytokine genes or arming with antitumor chemicals for tumoricidal therapy. Aim of the study was to:

Characterize peripheral blood mononuclear (PBM) and liver sinusoidal wash-out cells (LSWC) of normal and tumor bearing rats revealing predilection to *in vitro *adhere to the tumor tissue;

Investigate the proliferating liver tumor properties modifying *in vitro *adhesion of PBM and LSWC to the tumor foci.

## Methods

Male rats of 250ń300 g body weight (8ń9 weeks old) of the Wistar (W/Wag) strain were used. The CC531 is a 1,2-dimetylhydrazine-induced, moderately differentiated and weakly immunogenic adenocarcinoma of the colon, syngenic to WAG/Rij rats. The cells were kindly provided by Dr P. Kuppen, Leiden University Medical Center, Netherlands [7-9]. Liver tumors were induced by injection of 1 ń 10^6 ^CC531 colon cancer cells into the portal vein. Rat liver specimens were obtained 4 to 6 weeks after inoculation of CC531 cells. Specimens of 5 ń 5 ń 5 mm were snap frozen in liquid nitrogen and stored at -70ńC. Cryostat sections of 5 micrometers were cut at -20 degrees C and dried for 2 h. Liver sinusoidal washout cells (LSWC), were obtained by flushing livers of normal (LSWCn) and CC531 metastases bearing (LSWCt) rats through the portal vein with Hanks solution. The effluent fluid was collected. Peripheral blood mononuclear cells (PBM) were isolated from normal rats (PBMn) or rats with liver CC531 metastases (PBMt). The frozen sections were encircled with wax pen, diameter of 20 mm, to avoid spreading of the overlaid cells during agitation. Then 100 microliters of suspension containing 1,5 ń 10^6 ^of mononuclear cells were applied on sections and incubated under rotation for 30 min at 4 degrees C. The non adhering cells were rinsed off with cold PBS. The sections were labeled with mononuclear antibodies. To determine expression of surface molecules of adherent MNC immunohistochemical staining was performed using mAb anti: CD 11a, CD11b, CD18, CD54, CD49d, CD43, CD4, CD8, CD5, MHCII, CD14, CD56. Mononuclear cells which adhered to the sections stood as darkly stained cells above the plane of the tissue itself. Cells stained with monoclonal antibodies were recognized by their colored rim of membrane and cytoplasm [10].

## Results and Discussion

The CC531 liver metastases grow surrounded by leukocyte infiltrates. Moreover, leukocytes accumulate in tumor blood vessels. Is it a sign of anti tumor reaction or support for the proliferating tumor cells? Despite of the host leukocyte infiltrates the tumor mass increases in time. The question arises as to whether there is any adherence specificity of blood leukocytes to the CC531 tumor endothelial, epithelial and stromal cells? We have studied this problem comparing phenotypes of mononuclear cells marginating in the tumor-bearing liver in sinusoids and tumor vessels during an *in vivo *perfusion and subsequently washed-out, with: a) cells identified on histological sections and b) cells *in vitro *adhering to liver and tumor structures.

There were evidently more PBM adhering to tumor than liver tissue in terms of cell numbers. That difference in adherence rate to tumor foci was not as evident for LSWC. Evidently more both PBM and LSWC from normal and tumor bearing livers adhered to tumor stroma than epithelium.

In the PBMn population the CD14, CD8 and CD56 cells revealed predilection to adhere to tumor but also to a certain extent to liver tissue. The adhering CD14 cells were in the most part MHCII class negative. The CD5 population containing T cells showed weak adherence properties. PBMt revealed predilection of CD14, MHCII class positive cells. LSWCn adhered preferentially to tumor and liver mostly in the CD14 and MHCII populations. In case of LSWCt there were mostly MHCII positive cells adhering both to tumor and liver. PBMn expressing adhesion molecules CD11a, CD11b, CD18 and CD54 adhered avidly to tumor and liver. It was not found true for PBMt. LSWCn did not reveal any special trends, whereas LSWCt showed overall low adherence tendency due to low expression of adhesion molecules.

A phenotypical similarity was observed between cell populations washed-out from sinusoids of liver with metastases, cells found in tumor infiltrates on histology and cells adhering to the liver and tumor sections *in vitro*. The CD14+, MHC class II blood cells revealed predilection to adhere to both liver and CC531 metastases. Peripheral blood CD11a, CD11b, CD18 cells from normal but not tumor bearing-rats revealed strong tendency to adhere *in vitro *to tumor and liver. Liver sinusoidal blood CD11b cells from normal rats *in vitro *adhered to tumor and liver at a higher rate than cells from tumor bearing rat. There were no specific differences among the phenotypes of cells adhering to the tumor or liver.

## Concluding

Evidently more peripheral and sinusoidal blood cells *in vitro *adhered to the sites of CC531 than to the liver tissue. There were more of PBM than LSWC adhering to both liver and tumor. There seems to be unknown cell surface determinants responsible for a high rate of adherence of mononuclear cells to the tumor structures.

**Figure 1:**
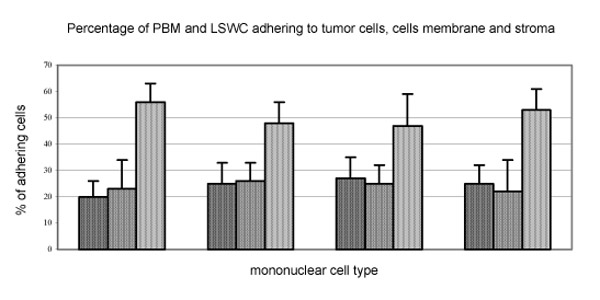
First bar represents percentage of cells adhering to tumor cells, second to tumor cells membrane, third to the tumor stromal structures.
